# Comparison of effects of age and sex on serum protein electrophoretic pattern in one-humped camels (*Camelus dromedarius*) in Semnan, Iran

**Published:** 2014-02-02

**Authors:** M. Ahmadi-hamedani, K. Ghazvinian, P. Kokhaei, M. Barati, A. Mahdavi

**Affiliations:** 1 *Department of Pathobiology, Faculty of Veterinary Medicine, Semnan University, Semnan, Iran*; 2 *Department of Animal Sciences and Food Industries, Faculty of Veterinary Medicine, Semnan University, Semnan, Iran*; 3 *Department of Immunology, Semnan University of Medical Sciences, Semnan, Iran*; 4 *Immune and Gene Therapy Lab. CCK, Karolinska University Hospital Solna, Stockholm, Sweden*

**Keywords:** Age, *Camelus dromedarius*, Electrophoresis, Semnan, Sex

## Abstract

The aim of this study was to evaluate the influence of age and sex on the concentration of total serum protein measured by the biuret method and protein fractions determined using cellulose acetate electrophoresis in apparently healthy camels (*Camelus dromedarius*). Blood samples were collected from 21 camels (12 males and 9 females). The camels were further divided into two groups: 12 young camels at the age of 3 months to 2 years and 9 adult camels at the age of 3-15 years. Cellulose acetate electrophoresis of serum proteins identified five protein fractions in adult camels as young camels, these five protein fractions include albumin, α1 and α2, β and γ-globulins. In adult camels, serum levels (g/l) of total protein, albumin, α1-globulins, α2-globulins, β-globulins and γ-globulins were 80.9±3.10, 42.9±3.10, 1.3±0.22, 2.2±0.30, 11.8±0.30 and 22.6±0.20, respectively. However, in young camels, these levels (g/l) were 66.8±2.90, 40.2±2.40, 1.0±0.14, 2.6±0.30, 10.6±0.80 and 12.3±1.20, respectively. The albumin/globulin (A/G) ratio was 2.08±0.28 in adult camels and 3.77±0.53 in young ones. The mean serum concentrations of total protein and γ-globulins were significantly (*P*<0.05) higher and the A/G ratio was significantly lower in adult camels compared to young camels. The mean concentrations of γ-globulins were significantly higher and the A/G ratio was significantly (*P*<0.05) lower in females compared to male camels. The results of the present study indicate a significant effect of age and sex on the concentrations of some of the serum protein fractions in dromedary camels.

## Introduction

A standard method for fractionation and quantification of serum proteins is electrophoresis in clinical biochemistry (Piccione *et al.*, 2011). Serum proteins are separated according to their electric charge using cellulose acetate electrophoresis (Alberghina *et al.*, 2010). Two major proteins are present in serum including albumin and globulins. Following serum protein electrophoresis, globulins are classified as alpha (α), beta (β), and gamma (ϒ) globulins. Also α- and β-globulins are subdivided into alpha 1 (α1)- and alpha 2 (α2)-, and beta 1 (β1)- and beta 2 (β2)-globulins. Each protein fraction excluding albumin, typically consists of two or more proteins (Tóthová *et al.*, 2013).

As the number of protein fractions varies with the species and with the type of support medium used (Keay and Doxey, 1981), determination of the normal electrophoretic pattern of each species appears to be essential. The Camelid serum protein electrophoretic patterns have been studied using cellulose acetate (Khadjeh, 1998), agarose gel (Chaudhary *et al.*, 2003) and capillary electrophoresis (Elkhair and Hartmann, 2011). Serum total protein and their variation with age and sex in Indian camels have been studied previously by Bhargava *et al*. (1964), Chartier *et al*. (1986) and Chiericato *et al*. (1986). However, only a few studies have described the effect of age and sex on these proteins and their electrophoretic pattern in dromedary camels (Khadjeh, 1998; Elkhair and Hartmann, 2011).

Accordingly the aim of this study is to evaluate the variation of total serum proteins and their electrophoretic pattern with age and sex in apparently healthy dromedary camels.

## Materials and Methods

This study was conducted on two age groups of apparently healthy camels (12 young camels and 9 adult camels) existing in four different herds (herd management and feeding were approximately similar) located in Semnan province of Iran. The 9 adult camels were between 3 to 15 years old including 4 males and 5 females. The 12 young camels were between 3 months to 2 years old including 8 males and 4 females. Blood samples (5 ml) for the analyses were collected from the jugular vein by vacutainer tubes without anticoagulant.

To evaluate total serum protein concentration and protein fractions, the blood serum was separated by centrifugation at 2,500 rpm for 10 min, and stored at −20°C until further analyses. Total serum protein concentrations of the samples were measured using biuret method using Pars Azmun commercial test kits (Tehran, Iran) and spectrophotometer (Biochrom WPA Biowave II) at wavelength 540 nm. Serum protein fractions were separated using electrophoresis on cellulose acetate plate in barbital buffer, pH 8.6, at 180 V, 4 mA, for 15 min according to manufacturer’s instructions (Helena Biosciences, UK).

After separation, the protein bands were stained with Ponceau S for 10 min, then destained with 5% acetic acid for 2 min and dehydrated in methanol for 2 min, and cleared with clearing solution (30% glacial acetic acid, 70% absolute methanol, and 4% clear aid) for 10 min, Finally, after drying at 50-60°C for 15 min, the relative levels of separated proteins were scanned using a densitometer at 525 nm. Protein fractions were identified and quantified by a computer software Phoresis (IRISPowerscan 9, Germany). The relative concentrations of the protein fractions were determined as the percentage of their optical absorbance. For calculating the absolute concentration, the percentage of each fraction in a serum sample was multiplied by the total protein concentration (Zamani Ahmadmahmudi *et al.*, 2012). Albumin to globulin (A/G) ratio was then calculated from the electrophoretic scan.

Data were analyzed using SAS software package (ver 9.1.3). The normal distribution of the data was evaluated using Kolmogorov and Smirnov test. A two sample t-test was used to compare the means between groups, and a P≤0.05 was considered to be significant.

## Results

**Fig 1:**
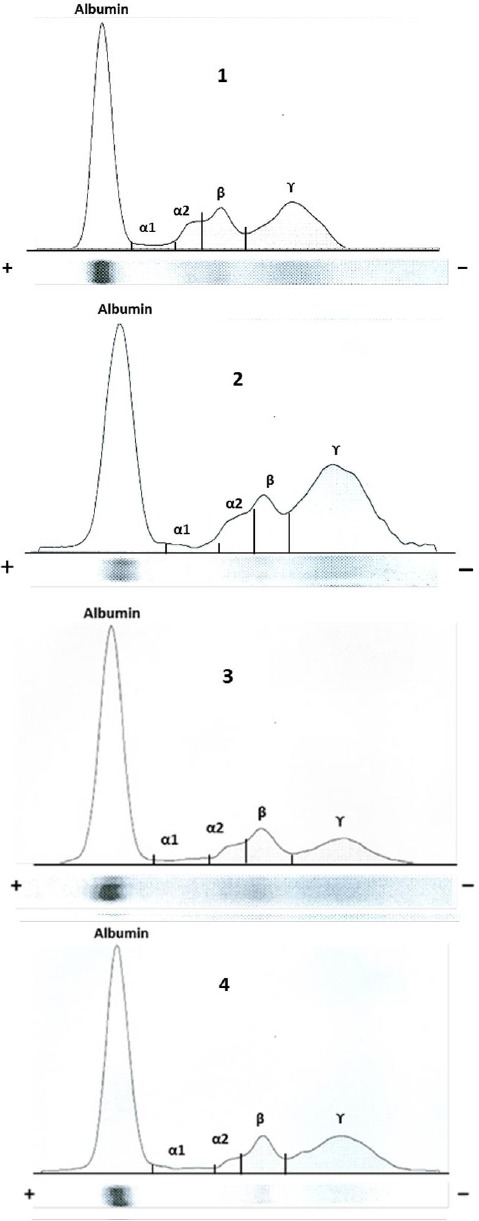
Electrophoretic pattern of serum proteins (albumin, and α_1_-, α_2_-, β- and γ-globulins) in young (1) and adult (2) camel, and male (3) and female (4) camel.

Cellulose acetate electrophoresis of serum proteins revealed five fractions: albumin, two α-globulins (α1 and α2), β-globulin and γ-globulin fractions in both sexes (female and male) and in young and adult camels. Electrophoretic patterns for serum from a young and an adult camel, a male and a female camel for comparison are shown in Fig. 1.

Concentrations (mean ± SD) of serum total protein and protein fractions in both age groups of camels are presented in Table 1.

**Table 1:** Serum concentrations (mean ± SD) of total protein (TP) and protein fractions in apparently healthy young (n=12) and adult (n=9) camels

Protein fractions	Group of Camels	
	Young	Adult
TP (g/L)	66.8±2.90**	80.9±3.10
Alb (g/L)	40.2±2.40	42.9±3.10
α_1_-Globulins (g/L)	1.0±0.14	1.3±0.22
α_2_-Globulins (g/L)	2.6±0.30	2.2±0.30
β-Globulins (g/L)	10.6±0.80	11.8±0.30
γ-Globulins (g/L)	12.3±1.20**	22.6±0.20
A/G ratio	3.77±0.53*	2.08±0.28

* (P<0.05) indicate significant differences between the young and adult camels.

** (P<0.01) indicate significant differences between the young and adult camels.

The mean concentrations of total protein (g/l) in young and adult camels were 66.8±2.90 and 80.9±3.10, respectively. Serum total protein concentration was significantly higher (*P*<0.01) in adults compared to young camels.

The concentrations of albumin (g/l) in young and adult camels were 40.2±2.40 and 42.9±3.10, respectively. This difference between young and adult camels was not statistically significant (P>0.05). The mean concentrations (g/l) of α1-, α2-, β-, γ-globulins and A/G ratio were 1.0±0.14, 2.6±0.30, 10.6±0.80, 12.3±1.20 and 3.77±0.53, respectively in young camels and 1.3±0.22, 2.2±0.30, 11.8±0.30, 22.6±0.20 and 2.08±0.28, respectively in adult camels. The mean concentrations of γ-globulins were significantly higher (*P*<0.01) in adult camels compared to young camels. The A/G ratio was significantly lower in adult camels than the young camels.

Concentrations (mean ± SD) of serum total protein and protein fractions in both sexes of camels are presented in Table 2.

**Table 2:** Serum concentrations (mean ± SD) of total protein (TP) and protein fractions in apparently female (n=12) and male (n=9) camels

Protein fractions	Group of Camels	
	Female	Male
TP (g/L)	76.3±4.40	70.2±3.00
Alb (g/L)	42.2±3.5	40.8±2.20
α_1_-Globulins (g/L)	0.94±0.15	1.3±0.22
α_2_-Globulins (g/L)	2.1±0.35	2.6±0.30
β-Globulins (g/L)	11.1±0.89	11.2±0.48
γ-Globulins (g/L)	19.9±1.80*	14.3±2.20
A/G ratio	2.21±0.23**	3.67±0.57

* (P<0.05)indicate significant differences between the female and male camels.

** (P<0.01) indicate significant differences between the female and male camels.

The mean concentrations (g/l) of total protein and albumin were 76.3±4.40 and 42.2±3.50, respectively in female, and 70.2±3.00 and 40.8±2.20, respectively in male camels.

No significant differences were found between female and male camels for total protein and albumin. The mean concentrations of α1-, α2-, β- and γ- globulins (g/l) and A/G ratio were 0.94±0.15, 2.1±0.35, 11.1±0.89, 19.9±1.80 and 2.21±0.23, respectively in females, and 1.2±0.18, 2.6±0.30, 11.2±0.48, 14.3±2.2 and 3.67±0.57, respectively in males. The mean concentrations of γ-globulins were significantly higher and the A/G ratio was significantly lower in females compared to male camels.

## Discussion

The current standard protocol for estimation of serum protein fractions in human (Vavricka *et al.*, 2009) and veterinary medicine (Chaudhary *et al.*, 2003; Zamani Ahmadmahmudi *et al.*, 2012; Tóthová *et al.*, 2013) is serum protein electrophoresis. Although this test has a low specificity in the diagnosis, measuring the normal serum protein electrophoresis patterns in all domestic animals and the correct interpretation of their results is very useful for the clinician in diagnosing healthy and infected animals (Lutz *et al.*, 2009).

Using cellulose acetate strips as a supporting matrix for the separation of serum proteins in comparison with other support media, has several advantages that include: lower absorption of albumin during electrophoresis, protein fractions can be separated more clearly, better staining and scan bands (Zamani Ahmadmahmudi *et al.*, 2012). By comparison to other animals, serum protein electrophoresis is rarely used as a diagnostic method for the evaluation of health status in camels. Therefore in the present study, total protein concentrations and protein electrophoretic patterns of dromedary camels were studied in two different age groups and in both sexes.

The mean total protein concentration in both groups of camels showed a statistically significant increase in adult camels than young ones. In other words, total protein concentration increases with age. This result is consistent with the results of Ghodsian *et al*. (1978), Chartier *et al*. (1986), Khadjeh (1998), Chaudhary *et al*. (2003) and Elkhair and Hartmann, (2011), but disagrees with the results reported by Kataria *et al*. (1991). Increased level of total protein concentration in adult camels than the young camels can be attributed to an increase in the concentration of albumin and γ-globulins (Chaudhary *et al.*, 2003).

In this study, cellulose acetate electrophoresis of serum proteins in camels revealed five fractions comprising albumin, α1 and α2, β, and γ-globulins and is in accordance with Mahendra and Ghosh (1961), AI-Ani *et al*. (1992), Khadjeh (1998) and Elkhair and Hartmann (2011) but in contrast to the report of Chaudhary *et al*. (2003) who reported six fractions. This difference can be attributed to the method used for electrophoresis. The mean albumin concentration in adult camels was higher than in young camels, but this increase was not significant. This finding is in line with data reported by Chaudhary *et al*. (2003), Elkhair and Hartmann, (2011) but does not agree with the results of Khadjeh (1998).

The results of our study indicate that levels of α1-globulins, β-globulins and γ-globulins increase and those of α2-globulins decrease with increasing age. The only higher levels of γ-globulins in adult camels than in the young ones were statistically significant.

Chaudhary *et al*. (2003) suggested that the immature lymphoid system causes the concentration of γ-globulins in young camels to be less than adult camels and these values remain low until globulins are generated by the maturing immune system. The A/G ratio is important to clinical pathologists, because it allows for a systematic classification of the electrophoretic profile and identification of dysproteinemias (Kaneko *et al.*, 2008).

In the present study the A/G ratio was significantly lower in adult camels than in young ones, as a result of higher globulin concentrations in adult animals. Similar results were reported by Alberghina *et al*. (2010) and Tóthová *et al*. (2013), but these results are not in agreement with that of Chaudhary *et al*. (2003).

The A/G ratio in both age groups of dromedary camels in the present study is wider than previous reports published by Chaudhary et al. (2003), Khadjeh (1998), Patodkar *et al*. (2010) and Elkhair and Hartmann, (2011), most likely because of the number of camels used or higher levels of albumin in this study compared to other mentioned studies.

The mean total serum protein and albumin levels in dromedary camels of both sexes showed a statistically non-significant increase in females than males. Khadjeh (1998) also reported a significantly higher total protein concentration in female camels than in male camels, however he indicated that the concentration of albumin was significantly higher in males than in female camels. The mean values of α-globulins (α1 and α2) and β-globulins in our research were slightly higher in males than in females, but the mean values of γ-globulins were significantly higher in females than in males.

In relation to the values of globulins in both sexes of camels, our results were consistent with the results of Khadjeh (1998) and Patodkar *et al*. (2010), but not with that of Elkhair and Hartmann, (2011). Higher levels of γ-globulins in female than male camels may be due to the effects of estrogen in females (Khadjeh, 1998).

The present study showed that the A/G ratio was significantly higher in males than in female camels. This result could be attributed to higher levels of γ-globulins in female camels. Similar results were reported by Khadjeh (1998) and Patodkar *et al*. (2010).

## Conclusion

In conclusion, serum protein electrophoresis and determination of absolute values of serum protein fractions in dromedary camels by cellulose acetate electrophoresis is very useful for clinicians to diagnose and evaluate various pathological conditions. The results presented in this study showed a significant effect of age and sex on the concentrations of some of the serum protein fractions in dromedary camels. The most significant age-related differences in this study included differences in the concentrations of serum total protein, γ-globulins and the A/G ratio between young and adult camels. In relation to the influence of gender, significant differences were observed in the concentrations of γ-globulins and the A/G ratio between male and female camels. The results of the current study could provide a basis for more extensive studies with regard to the use of cellulose acetate electrophoresis for serum proteins in dromedary camels.
